# Structural, Dermal and Ungual Characteristics of the Foot in Patients with Type II Diabetes

**DOI:** 10.3390/medicina55100639

**Published:** 2019-09-25

**Authors:** Cristina Gonzalez-Martin, Sonia Pertega-Diaz, Teresa Seoane-Pillado, Vanesa Balboa-Barreiro, Alfonso Soto-Gonzalez, Raquel Veiga-Seijo

**Affiliations:** 1 Research Group of Clinical Epidemiology and Biostatistics, Biomedical Research Institute of A Coruña (INIBIC), University Hospital Complex of A Coruña (CHUAC), SERGAS, University of A Coruña, 15006 A Coruña, Spain; sonia.pertega.diaz@sergas.es (S.P.-D.); maria.teresa.seoane.pillado@udc.es (T.S.-P.); vanesa.balboa.barreiro@sergas.es (V.B.-B.); raquel.veiga.seijo@udc.es (R.V.-S.); 2 Endocrinology Service of the University Hospital Complex of A Coruña (CHUAC), SERGAS, University of A Coruña, 15006 A Coruña, Spain; alfonso.soto.gonzalez@sergas.es

**Keywords:** foot, diabetes, podiatry

## Abstract

*Background and Objectives:* Diabetes is a chronic and metabolic disease, considered as an important public health problem. The objective of this study was to determine the prevalence of podiatric pathology in type II diabetic patients. *Materials and Methods:* An observational descriptive study of prevalence in the endocrinology service of Complexo Hospitalario Universitario A Coruña (CHUAC) (A Coruña-Spain) was carried out (*n* = 153). Type II diabetic patients included, of legal age who signed the informed consent. Sociodemographic variables were studied (age, sex, body mass index (BMI), smoking habit, alcohol consumption, family history), disease variables (time of evolution of diabetes, treatments, low-density lipoprotein (LDL), high-density lipoprotein (HDL), glucose), podiatric variables: measurement of the footprint, metatarsal and digital formula, nail, skin, hindfoot and forefoot alterations. The data collection was done in 2018 and the data analysis was carried out in 2019. *Results:* The patients with type II diabetes had greater age, obesity and arterial hypertension it compared to the general population. Diabetic patients had a higher prevalence of flat feet than the general population (71.2% vs. 20.7%, *p* < 0.001), with a predominance of normal foot according to the podoscope. The predominant podological pathology was the presence of claw toes (94.8%), followed by dermal (78.4%) and nail (71.9%) alterations, and the Hallux Valgus (66.0%). The Clarke angle and the Chippaux index showed a Kappa concordance index of 0.26 with the type of footprint measured with the podoscope. The Staheli index showed a Kappa index of 0.27 associated with an observed agreement of 54%. *Conclusions:* This study shows that foot problems continue to be prevalent in subjects with type II diabetes mellitus and for this reason, podiatry is essential in its treatment.

## Highlighting

Updated information on podiatric pathology data in patients with type II diabetes mellitus. These data can help to establish prevention criteria in clinical practice.

## 1. Introduction

Type II diabetes is increasing worldwide due to the current sedentary lifestyle, high obesity and longer life expectancy [1].

Diabetes is a chronic disease that presents a high morbimortality due to the complications that develop during the evolution of the disease [2].

In Spain, the prevalence of diabetes mellitus (DM) is estimated at 9.4% (10.6% men, 8.2% women) [3]. The most important risk factors for DM are age, obesity and family history of DM. The prevalence of the different chronic complications varies depending on the type of DM, the time of evolution of the disease and the degree of metabolic control, with an estimate of 25% of neuropathy, 32% of retinopathy and 23% of nephropathy [4].

Diabetes can cause serious complications at the level of the foot, among these complications is diabetic neuropathy (loss of normal nervous function) that affects 40% of this population [5] and peripheral vascular disease (loss of normal circulation) [6].

Diabetic neuropathy and vascular diseases are usually present in many diabetic patients. Neuropathy is associated with metabolic abnormalities of diabetes and will cause insensibility and deformities in the foot, which usually occur with a gait pattern with alterations. By joining the diabetic neuropathy, peripheral vascular disease will cause any external pressure or friction of footwear on the foot and can lead to an injury that may end in ulceration. The most frequent areas of ulceration will be the fingers, the heel, and the bones of the ankle [6,7].

84% of ulcers that do not heal lead to amputations of the lower extremities of diabetic patients. The frequency of mortality of diabetic patients after a major amputation varies from 11 to 41% in the first year [6,7,8].

DM is one of the diseases with the greatest sociosanitary repercussions, not only because of the frequency of this disease, but also because of the impact of the complications with which these patients attend, as it happens with the feet. In this way, the complications in the feet affect to quality of life, social participation and livelihood [1,8,9].

The fact of knowing the main foot problems can help to prevent and treat diabetic foot and avoid serious complications such as amputations [7]. For all the above and the absence of relevant studies on pathology at the level of the foot, the objective of this work was to determine the prevalence of podiatric pathology in type II diabetic patients.

## 2. Materials and Methods

### 2.1. Design and Field of Study

An observational descriptive study of prevalence was carried out. It was carried out in the Service of endocrinology and in the Clinical Epidemiology and Biostatistics Unit of the University Hospital Complex of A Coruña (CHUAC) (A Coruña, Spain). This study is part of a broader multidisciplinary study with endocrine, vascular, digestive, ophthalmologists and nursing staff (*n* = 505). From the sample of patients included in the study (*n* = 505), a subsample was taken (*n* = 153) where the podiatry scan was performed. The general and chiropody characteristics of these patients were compared with a randomized population sample of the same geographical area (*n* = 1844). The foot examinations of the patients included in the study were performed by podiatrists.

The data collection was done in 2018 and the data analysis was carried out in 2019.

### 2.2. Inclusion and Exclusion Criteria of the Sample Studied

Of the patients included, all of them type II diabetics, who were of legal age and who gave their informed consent in writing. Type I diabetic patients with a history of cardiovascular and hepatic disease were excluded, to ensure that the foot problems are focused on the diabetic disease and avoid bias in the results. All the people included in the study went to the endocrinology clinic and the characteristics of the study were explained to them. Those interested in participating were summoned another day to sign the informed consent and to perform the explorations.

### 2.3. Variables Studied and Procedure

The following variables were collected from each person included in the study: sociodemographic variables (age, sex, Body Mass Index (BMI), smoking habit, alcohol consumption, and family history), disease variables (time of evolution of diabetes, pharmacological treatments, low-density lipoprotein (LDL), high-density lipoprotein (HDL), glucose), podiatric variables: measurements of the footprint through podoscopy and pedigraphy. To study the footprint by pedigraph, three footprint measurements were used: Clarke’s angle, Chippaux–Smirak and Staheli index [10]. These parameters are usually used to categorize the footprint as cavus, flat or normal foot. The footprints were obtained by placing a reticulated piece of rubber sheeting, tensed and impregnated with ink, between the subject’s foot and a piece of stretched paper. In order to get an accurate footprint, it was performed while the participants were sitting. They were also studied metatarsal and digital formula, nail changes, dermal, of the hindfoot and forefoot, the absence of hair, skin color and temperature and the presence of edema were also determined. The participants were on a stretcher to collect this data.

### 2.4. Ethical and Legal Aspects

The study is approved by the ethics committee of Galicia (CAEIG 2016/72; approval date: 25 May 2016). Furthermore, ethical and legal aspects were considered in this study. All participants were informed of the objective of the study and its procedure. Informed consent was necessary to participate in the study.

### 2.5. Statistical Analysis

A descriptive analysis of the variables included in the study was carried out, the quantitative variables were expressed as mean ± standard deviation (SD) and the qualitative variables as frequency (n) and percentage.

We compared the random sample with the general population, in turn, we compared the podological characteristics of the sample studied according to sex. For the comparison of means, the Student’s *t*-test or Mann–Whitney U test was used depending on the nature of the variables. The association between qualitative variables was analyzed using the Chi-square test or Fisher’s test.

The concordance between the different image diagnoses and the diagnosis by means of a podoscope were analyzed through the Kappa concordance index.

## 3. Results

### 3.1. Sociodemographic Characteristics of the Sample Studied

We analyzed 505 patients diagnosed with type II diabetes, whose general characteristics and comorbidity (Table 1) were compared with the characteristics presented in the healthy general population sample belonging to a previous study [11].

The sample of diabetic patients presented a mean age of 62.9 ± 7.9 years, higher than the age presented by the general population. Diabetic patients had a higher prevalence of obesity (56.9% vs. 39.2%) and HBP (42.4% vs. 36.5%) than the general population. Regarding smoking, there was a greater predominance of smokers in the general population (17.5% vs. 2.6%), however, the same distribution was determined according to sex in both samples.

### 3.2. Podiatric Alterations

Podiatry exploration was carried out in a subsample of 153 diabetic patients (Table 2). Patients with diabetes had a higher prevalence of flat foot than patients in the general population (71.2% vs. 20.7%, *p* < 0.001), among whom normal foot prevailed, according to the podoscope.

More than 60% of the 153 diabetic patients presented normal foot according to the Clarke angle and the Chippaux index (60.4% and 61.9% respectively), whereas the Staheli index determined a higher prevalence of flat foot (38.8%) followed by a 30.6% normal foot. These classifications were similar in terms of the sex of the patients.

#### 3.2.1. Podiatric Alterations Depending on Sex

Table 3 shows the podiatric characteristics of the 153 patients diagnosed with diabetes according to sex. It was found that 66% had Hallux Valgus, the prevalence being slightly higher in women (69.5% vs. 62%, *p* = 0.326). 94.8% of the patients presented claw toes, presenting a similar prevalence in both sexes. 71.9% presented nail disorders, the most prevalent being onychocryptosis (55.7%). 78.4% presented dermal alterations (xerosis 25.5% and hyperkeratosis 64.1%), more frequent among women (86.6% vs. 69%, *p* = 0.008). Forty-four percent of the patients presented abnormal coloration in the skin of the lower extremities, being in this case more frequent in men (52.9% vs. 35.6%, *p* = 0.038). In contrast, a higher prevalence of edema was observed in females (39.0% vs. 22.5%, *p* = 0.028).

#### 3.2.2. General Characteristics According to the Type of Footprint and the Podiatric Alterations

According to the type of footprint (Table 4), a higher BMI and abdominal perimeter was found among patients with flat feet, as well as an increase in the presence of hyperkeratosis. The time of evolution of the disease is slightly higher among patients with normal footprint, without 2nd toe claw or without Hallux Valgus, without significant differences being observed. In turn, patients with 2nd claw finger or Hallux Valgus had a higher prevalence of hyperkeratosis (64.4% and 67.3% respectively).

#### 3.2.3. Concordance Between Footprint According to the Podoscope

The concordance between the type of footprint according to the podoscope and the measurements using the Clarke angle and the Chippaux and Staheli indexes was analyzed. A greater agreement percentage was observed between the footprint type according to the podoscope and the Staheli index (54%), followed by the Chippaux index (50%) and, to a lesser extent, the Clarke angle (49%). A Kappa index between 0.26 (Chippaux index and Clarke angle) and 0.27 (Staheli index) was observed.

## 4. Discussion

This work constitutes a research that tries to cover the gaps of knowledge found in the literature during the course of this work in Spain with a podiatric perspective, in which there has been a lack of studies that tried to know the repercussion that type II diabetes mellitus triggers at the level of the foot.

Although we find in the literature different studies that tried to know the prevalence of diabetic foot, and different alterations in the foot in people with this condition [12,13,14], we wanted to study the impact in a broad way, knowing the repercussions at all levels of the foot: structural, dermal and nail aspects.

Taking into account that among the risk factors for diabetes are age [15] and obesity [16,17], our sample has a high age (62.9 ± 7.9 years) with a minimum age of around 40 years compared with other studies found in the literature [18,19], likewise, we are struck by the high obesity present in our study, a fact that is higher than the literature consulted [19].

In the present study we found a high presence of podiatric pathology, finding 94.8% of claw fingers, data that are higher than that found in the general population as well as in the diabetic population (9%) [17]. In relation to the deformity of Hallux valgus, we found in our study a prevalence of 66%; we have not found any study with data on this pathology.

Alavi et al. and Vural et al. [19,20] studied nail changes as in our study, finding similar data on onychodystrophy (13% in our study vs. 11% in the study by Vural et al. [19]). On the other hand, we found a higher prevalence of onychogyphosis in our study (12.9% vs. 4% in the study by Vural et al. 19). The results obtained in relation to onycholysis (2.9%) were lower than those found in the literature [19,20].

Regarding the skin alterations, we found studies that dealt with xerosis and hyperkeratosis. Regarding xerosis, we found a lower prevalence in this work compared to the literature reviewed [18,19]. On the other hand, the prevalence of hyperkeratosis was higher in this study, compared to the data consulted [19,21,22,23].

In relation to the measurements in footprints, Plumarom et al. [24] discovered that the Staheli index could be considered as the screening or diagnostic method for flatfoot. In our study, the major of the footprints corresponded with flatfoot. Furthermore, we analyzed the concordance between the methods with the podoscope, and we got the best agreement between the podoscope and the Staheli Index.

Based on the findings of this study, it is possible to observe the importance that type II diabetes mellitus triggers at the level of the lower limb, revealing the need for adequate podiatric prevention [25] in patients with this pathology in collaboration with a multidisciplinary team.

### Limitations

The results of the present study should be interpreted taking into account their possible limitations. A potential problem with this study is that no data were collected from patients who refused to participate, so the sample may not be representative of the population with diabetes. However, the percentage of patients who declined to participate in the study was less than 5%, so it can be considered that the representativeness of the sample has not been affected.

Regarding potential information biases, although they cannot be totally ruled out, several strategies have been followed to avoid them. On the one hand, to minimize possible bias of the observer, the podiatric study has been carried out by two podiatrists who have agreed on the findings. On the other hand, the measurement of subjective concepts that can be perceived differently by different people, such as pain, functionality or health related to the foot, can be sources of bias. The use of validated questionnaires such as those used in this study limits this problem, and also makes it possible to compare the findings obtained with those of other similarly themed publications.

## 5. Conclusions

This study concluded with findings including a high prevalence of podiatric pathology in diabetic patients, mainly flat feet, hallux valgus and claw toes. We found a predominance of skin alterations in female patients, whereas abnormal color in the feet was more frequent in the male patients.

In order to substantiate the concordance between the type of footprint according to the podoscope and the measurements studied in the footprint, the Staheli index showed a greater percentage according to the type of footprint according to the podoscope than the Chippaux index and the Clarke angle, showing a weak concordance in all three cases.

## Figures and Tables

**Table 1:** General characteristics and comorbidity of diabetic patients, and comparison with those of healthy general population (*).

	General Population (*n =* 1844)	Diabetic (*n =* 505)	
	Mean (SD)	Mean (SD)	*p*
**Age (years)**	61.8(12.4)	62.9(7.9)	**0.016**
BMI (Kg/m^2^)	29.2(4.7)	31.7(5.4)	**<0.001**
Abdominal perimeter (cm)	95.4(12.7)	105.6(13.8)	**<0.001**
SAT	131.4(17.2)	82.2(10.1)	**<0.001**
DAT	75.2(10.3)	141.1(17.7)	**<0.001**
LDL	132.0(31.4)	101.3(30.2)	**<0.001**
Glucose	99.2(26.6)	136.6(44.04)	**<0.001**
Diabetes evolution time (years)		12.9(8.6)	
	***n (%)***	***n (%)***	
**Sex**			0.995
Men	840(45.7)	231(45.7)	
Woman	997(54.3)	274(54.3)	
**BMI**			**<0.001**
Normal weight	327(17.9)	41(8.2)	
Overweight	784(42.9)	172(34.6)	
Obesity	717(39.2)	283(56.9)	
**Smoking habit**			**<0.001**
No	1008(55.0)	488(96.6)	
Former smoker	505(27.6)	4(0.8)	
Yes	320(17.5)	13(2.6)	
**Consumption of alcohol**			
No		497(98.4)	
Casual drinker		6(1.2)	
Chronic drinker		2(0.4)	
**Hypertension**			**0.018**
No	1155(63.5)	291(57.6)	
Yes	663(36.5)	214(42.4)	
**Treatments**			
Insulin	35(1.9)	111(22.0)	**<0.001**
Oral antidiabetics	174(9.6)	406(80.4)	**<0.001**
Diet		297(63.6)	
Exercise		287(61.2)	

BMI: body mass index, SAT: systolic blood pressure, DAT: diastolic blood pressure, LDL: light density lipoproteins. Numbers written in bold indicate statistically significant differences.

**Table 2:** Description and concordance of the footprint type according to podoscope and image.

	General Population (*n =* 1844)	Diabetic * (*n =* 153)		Diabetic * by Sex		
				Man (*n =* 71)	Woman (*n =* 82)	
	*n (%)*	*n (%)*	*p*	*n (%)*	*n (%)*	*p*
**According to podoscope:**			**<0.001**			0.260
Cavus	336(18.9)	21(13.7)		12(16.9)	9(11.0)	
Normal	1075(60.4)	23(15.0)		13(18.3)	10(12.2)	
Flat	369(20.7)	109(71.2)		46(64.8)	63(76.8)	
**Metatarsal formula**			**0.009**			0.403
Index Plus	241(24.1)	20(13.1)		12(16.9)	8(9.8)	
Index Plus-minus	349(35.0)	63(41.2)		27(38.0)	36(43.9)	
Index Minus	407(40.9)	70(45.8)		32(45.1)	38(46.3)	
**Digital formula**			0.058			0.403
Egyptian	565(57.1)	90(58.8)		45(63.4)	45(54.9)	
Greek	311(31.4)	55(35.9)		22(31.0)	33(40.2)	
Square	113(11.4)	8(5.2)		4(5.6)	4(4.9)	
**According to image:**						
**Clarke Angle**			**<0.001**			0.109
Cavus	434(27.5)	10(7.5)		7(11.7)	3(4.1)	
Normal	892(56.5)	81(60.4)		38(63.3)	43(58.1)	
Flat	254(16.1)	53(39.6)		15(25.0)	28(37.8)	
**Chippaux Index**			**<0.001**			0.084
Cavus	148(9.4)	12(9.0)		9(15.0)	3(4.1)	
Normal	658(41.6)	83(61.9)		34(56.7)	49(66.2)	
Flat	774(49.0)	39(29.1)		17(28.3)	22(29.7)	
**Staheli Index**			**<0.001**			0.297
Cavus	385(24.4)	41(30.6)		22(36.7)	19(25.7)	
Normal	249(15.7)	41(30.6)		15(25.0)	26(35.1)	
Flat	947(59.9)	52(38.8)		23(38.3)	29(39.2)	

* Subsample of diabetic patients in which podiatric examination was performed. Numbers written in bold indicate statistically significant differences.

**Table 3:** Podiatry characteristics of diabetic patients.

	Diabetic * (*n* = 153)	Diabetic * by Sex		
		Man (*n* = 71)	Women (*n* = 82)	
	*n (%)*	*n (%)*	*n (%)*	*p*
**Forefoot alterations**				0.634
No	149(97.4)	69(97.2)	80(97.6)	
Yes	4(2.6)	2(2.8)	2(2.4)	
**Hallux Extensus**	38(24.8)	20(28.2)	18(22.0)	0.375
**Hallux Valgus**	101(66.0)	44(62.0)	57(69.5)	0.326
**Grades**				0.662
Grade 1	47(46.5)	23(52.3)	24(42.1)	
Grade 2	30(29.7)	12(27.3)	18(31.6)	
Grade 3	13(12.9)	4(9.1)	9(15.8)	
Grade 4	11(10.9)	5(11.4)	6(10.5)	
**Claw fingers**	145(94.8)	68(95.8)	77(93.9)	0.725
2nd	132(86.3)	62(87.3)	70(85.4)	0.726
3rd	126(82.4)	61(85.9)	65(79.3)	0.282
4th	136(88.9)	66(93.0)	70(85.4)	0.136
**Nail disorders**				0.731
No	43(28.1)	19(26.8)	24(29.3)	
Yes	110(71.9)	52(73.2)	58(70.7)	
**Onychocryptosis**	78(55.7)	34(49.3)	44(62.0)	0.131
**Onychogyphosis**	18(12.9)	9(13.2)	9(12.5)	0.897
**Onychodystrophy**	18(13.0)	7(10.0)	11(16.2)	0.205
**Onycholysis**	4(2.9)	2(2.9)	2(2.9)	0.679
**Dermal alterations**				0.008
No	33(21.6)	22(31.0)	11(13.4)	
Yes	120(78.4)	49(69.0)	71(86.6)	
**Xerosis**	39(25.5)	17(23.9)	22(26.8)	0.683
**Hyperkeratosis**	98(64.1)	40(56.3)	58(70.7)	0.064
**Absence of hair**				0.494
No	86(56.2)	42(59.2)	44(53.7)	
Yes	67(43.8)	29(40.8)	38(46.3)	
**Skin coloring**				**0.038**
Normal	79(56.0)	32(47.1)	47(64.4)	
Anormal	62(44.0)	36(52.9)	26(35.6)	
**Skin temperature**				0.173
Normal	119(83.8)	54(79.4)	65(87.8)	
Anormal	23(16.2)	14(20.6)	9(12.2)	
**Edema**				**0.028**
No	105(68.6)	55(77.5)	50(61.0)	
Yes	48(31.4)	16(22.5)	32(39.0)	

* Subsample of diabetic patients in which podiatric examination was performed. Numbers written in bold indicate statistically significant differences.

**Table 4:** Characteristics of diabetic patients according to the type of footprint and the podiatric alterations: 2nd claw toe and Hallux Valgus.

	Type of Footprint			
	Mean (SD)	Mean (SD)	Mean (SD)	*p*
	Normal	Cavus	Flat	
**Age(years)**	61.4(9.0)	64.7(6.6)	62.8±8.3	0.422
**BMI (Kg/m2)**	28.4(3.4)	26.7(3.1)	32.2(5.3)	**<0.001**
	***n (%)***	***n (%)***	***n (%)***	
**Sex**				0.260
Men	13(56.5)	12(57.1)	46(42.2)	
Woman	10(43.5)	9(42.9)	63(57.8)	
**Time of evolution (years)**	11.7(6.9)	19.6(10.3)	12.6(8.5)	**0.013**
**Abdominal perimeter (cm)**	99.7(10.5)	97.5(10.1)	108.7(14.5)	**0.001**
**Hyperkeratosis**				**0.006**
No	14(60.9)	10(47.6)	31(28.4)	
Yes	9(39.1)	11(52.4)	78(71.6)	
	**2nd claw toe**			
	**No**	**Yes**		***p***
**Age(years)**	61.8(10.7)	63.0(7.8)		0.985
**BMI (Kg/m2)**	30.7(5.79)	30.9(5.2)		0.674
	***n (%)***	***n (%)***		
**Sex**				0.726
Men	9(42.9)	62(47.0)		
Woman	12(57.1)	70(53.0)		
**Time of evolution (years)**	16.2(7.2)	13.0(9.1)		0.054
**Abdominal perimeter (cm)**	104.0(14.0)	106.0(14.2)		0.676
**Hyperkeratosis**				0.825
No	8(38.1)	47(35.6)		
Yes	13(61.9)	85(64.4)		
	**Hallux Valgus**			
	**No**	**Yes**		***p***
**Age (years)**	62.1(9.8)	63.2(7.3)		0.603
**BMI (Kg/m2)**	31.3(5.5)	30.6(5.1)		0.461
	***n (%)***	***n (%)***		
**Sex**				0.326
Men	27(51.9)	44(43.6)		
Woman	25(48.1)	57(56.4)		
**Time of evolution (years)**	14.6(8.3)	12.9(9.1)		0.109
**Abdominal perimeter (cm)**	106.3(14.2)	105.5(14.1)		0.679
**Hyperkeratosis**				0.239
No	22(42.3)	33(32.7)		
Yes	30(57.7)	68(67.3)		
**Onychocryptosis**				0.882
No	19(45.2)	43(43.9)		
Yes	23(54.8)	55(56.1)		

BMI: body mass index. Numbers written in bold indicate statistically significant differences.
